# Supplementary material for: Are carbon emissions trading and green financial instruments synergistic? -Comprehensive quantitative research based on content analysis
Source: PLoS One. 2024 Mar 7;19(3):e0298601. doi: 10.1371/journal.pone.0298601 (PMC10919601; doi:10.1371/journal.pone.0298601)
Supplement: S1 Data — (ZIP) [file pone.0298601.s001.zip › Data/地级市Z_F工作报告.zip!/╔╜╬≈/╦╖╓▌╩╨/└┤╫╘╣1⁄2╓┌║┼í╛┬φ┐╦╩2╛▌═°í┐_2015.docx]

各位代表：

现在，我代表市人民政府向大会报告工作，请予审议，并请市政协委员和其他列席人员提出意见。

一、2014年工作回顾

过去一年，全市发展遇到前所未有的挑战，经济形势复杂严峻，下行压力持续加大，各种困难相互交织。在市委的坚强领导下，我们认真贯彻落实党中央国务院稳增长促改革调结构惠民生政策措施和省委省政府决策部署，保持推进“两大任务”工作定力，迎难而上，奋力作为，统筹抓好经济增长和结构优化，着力推进改革开放和创新驱动，大力推动发展质量和人民生活水平提升，全力维护社会和谐稳定，全市经济社会发展在克服困难中取得新成效，迈出新步伐。

(一)经济运行总体平稳。坚持把稳增长放在首要位置，采取了一系列措施。千方百计稳定煤电运行。贯彻落实省政府“煤炭20条”和“煤炭17条”，出台《关于化解煤矿及涉煤企业经营困难实施办法》《确保火电企业稳定运行六条措施》，清理规范涉煤收费，煤炭生产保持稳定，发电量用电量保持增长。全年生产原煤2.03亿吨；发电310亿度，增长10.6%；全社会用电105.3亿度，增长2.8%。狠抓重点工程建设。扎实开展“项目见效年”活动，重点工程“六位一体”六项指标全部超额完成省定任务，省市重点工程完成投资714.5亿元。精准帮扶企业。出台《关于帮扶非煤电工业企业发展六条措施》，设立循环经济发展专项资金、现代农业专项扶持资金、工业项目专项扶持资金，支持非煤电困难企业技术改造，奖励和补助节能降耗，减轻了企业负担。

全年地区生产总值完成1003.4亿元，增长4.5%(按可比价计算)；工业增加值完成443亿元，增长4.5%(按可比价计算)；固定资产投资完成815.3亿元，增长5.2%；社会消费品零售总额完成258.3亿元，增长12.4%；公共财政收入完成86.6亿元，下降9.1%；城镇常住居民人均可支配收入达到25725元，增长7.7%；农村常住居民人均可支配收入达到10137元，增长10.8%。

(二)产业结构调整取得积极进展。大力推进煤电基地建设。建成标准化矿井40座，标准化矿井建设进度、一级矿井数量位于全省前列。大力发展低热值煤发电、风力发电、光伏发电、生物质能发电项目，电力装机容量达到786.6万千瓦。在建发电项目26个，装机容量258万千瓦。积极发展循环经济。工业固废综合利用产业化取得新突破，固废工业园区进一步壮大，平朔粉煤灰综合利用项目首批白炭黑产品调试成功，工业固废年消化量达到3000万吨，综合利用率达到61%。成功举办亚洲粉煤灰综合利用技术国际交流大会。积极推进晋北现代煤化工基地前期工作。我市被国家发改委确定为资源综合利用“双百工程”示范基地，被工信部确定为工业绿色转型试点城市。加快发展新兴产业。坚持市直部门与县区结对子引进建设投资5亿元以上转型项目。新兴产业完成投资130亿元，同比增长25%，占全市工业投资的31%。旅游业总收入达到109.3亿元，增长29.9%。实施创新驱动发展战略。设立企业技术研发创新项目专项资金，申报成功煤电污染控制及废弃物资源化利用山西省重点实验室。加大日用陶瓷等行业研发投入，新增企业技术中心23家，各类技术中心达到70家；新增高新技术企业7家，高新技术企业达到11家。

(三)“三农”工作成效显著。加大强农惠农政策力度。大力推进农民户均增收万元工程，各级财政投入“三农”资金达19.96亿元，农民收入突破万元大关。着力提高农业综合生产能力。治理水土流失面积32万亩，新增节水面积6万亩，农田实灌面积达到204.7万亩。全年粮食产量达到25.6亿斤，比上年增长8.9%，增幅居全省第一。大力发展特色现代农业。制定实施《雁门关生态畜牧经济区领头雁工程和核心区建设规划》，应县恒天然奶牛牧场群两个大牧场投入使用，规模健康养殖迈上新台阶，奶牛存栏量达到18.1万头，鲜奶产量达到51.9万吨，肉羊年出栏量达到350万只，人均畜产品年占有量达到147公斤，当年农民人均畜牧业纯收入达到1400元，畜牧业五项指标继续保持全省第一。加快推进农业产业化。积极扶持农业产业化龙头企业，全市农产品加工企业完成销售收入160亿元，增长22%。大力推进农机社会化服务，农作物综合机械化水平达到68.2%。着力改善农村人居环境。扎实推进完善提质、农民安居、环境整治、宜居示范四大工程，全市完成投资2.5亿元。继续办好农村“五件实事”，改造农村困难家庭危房7100户，改造农村幼儿园14所，易地搬迁农村贫困人口3692人，深入推进乡村清洁工程，培训新型职业农民1万人。为全市农户免费发放冬季取暖用煤43.85万吨。实施扶贫攻坚，减少贫困人口20046人。

(四)城镇化水平稳步提高。深入推进“五城联创”，成功创建国家园林城市。加强中心城市建设。推进七里河市区段综合治理和城市棚户区、城中村改造，完成城中村拆迁47.5万平方米。继续推进朔州老城改造。大力推进热电联产集中供热，关停27台区域燃煤锅炉，热电联供供热面积新增500万平方米，达到1800万平方米，占供热总面积的81%。积极建设特色城镇。全市开工建设各类保障性住房12866套，完成投资43.6亿元。金沙滩、凤凰城、神头等八个镇被评为全国重点镇。全市城镇化率达到52.1%，比上年提高1个百分点。持续推进生态建设。积极开展植树造林，营造林33.1万亩，超省考核任务16个百分点。强化节能减排，狠抓大气污染防治，全年万元地区生产总值综合能耗同比下降3.65%，主要污染物减排任务全部完成。加强生态治理修复，投入5000多万元，推进采煤沉陷区治理试点工作。

(五)民生改善取得新成果。全市财政在民生领域投入111.9亿元，占到公共财政预算支出的78.9%。实施积极的就业政策。持续推进国家级创业型城市建设，新增城镇就业人数2.41万人。完善社会保障体系。扩大社会保险覆盖面，提高城镇居民、城镇职工医疗保险待遇，我市被国家人社部列为全民参保登记计划试点城市、社会保障卡综合应用试点城市和持卡人基础数据库建设试点城市。积极发展教育事业。进一步改善学前教育条件。继续推进义务教育阶段学校标准化建设。加快发展高等教育，中北大学朔州校区招生规模进一步扩大，在校学生达到3000人。着力提高基本公共卫生服务保障水平。深化医药卫生体制改革，稳步推进县级公立医院改革。人均基本公共卫生服务经费由每人30元提高到35元。在全市新农合定点医疗机构实行“先住院后付费”，有效减轻了患者就医负担。发展文化体育事业。扎实推进国家公共文化服务体系示范区创建工作，公共文化服务能力得到加强。积极开展体育运动，举办了朔州市第二届全民运动会。竞技体育实现新突破，第十四届省运会上成绩显著，位次前移。

为民兴办的十件实事全部落实。新改扩建公办标准化幼儿园12所；实施学生饮用奶全覆盖工程，10.6万寄宿学生喝上饮用奶；采取政府购买公共服务等方式，为高校毕业生提供了1012个就业岗位、组织了3100名毕业生参加就业实习；实施新农保和城居保两项制度并轨，城乡居民基础养老金月标准由65元提高到95元；提高农村五保户供养标准，平均每人每年由原先的分散供养2119元和集中供养2802元，分别提高到2708元和3791元；实现太原、大同两个我市转外就医最集中地区异地就医即时结算报销；提供玉米、奶牛、森林等农业政策性保险；实施饮水解困工程，解决了24个村、8300人的饮水困难；建成100个副食品生产基地惠民直销店；送戏下乡1231场。十件实事给广大群众带来实实在在的好处，受到群众称赞。

(六)改革开放迈出新步伐。认真落实转型综改试验2014年行动计划，着力创新体制机制。转型综改工作考核位居全省第一。深化行政体制改革。稳步推进市县政府机构改革。积极推进行政审批制度改革，取消或部分取消行政审批事项13项，下放行政审批事项24项，市本级保留行政审批事项222项，数量在全省最少。稳步推进农村改革。深化农村改革试验区建设，土地承包经营权确权登记颁证试点工作有序推进。创新地方金融发展机制。启动农村信用社改革，山阴县农信社改制方案获国家银监会批准。加快发展资本市场，两家企业在上海股权托管中心挂牌。加强融资服务平台建设，每季度组织一次银企对接会。加强招商引资，到位外来资金714.56亿元。

(七)安全稳定工作得到加强。狠抓安全生产。认真落实“党政同责、一岗双责、齐抓共管”制度和两个主体责任，深入开展安全生产大检查,全年共发生各类生产经营性事故198起，同比减少57起，下降22.35%，未发生较大及以上事故；死亡35人，同比减少4人，下降10.26%，安全生产工作考核位居全省第一，安全生产形势持续稳定好转。着力解决涉及群众切身利益的问题，公交、出租、企业改制等一些难点问题得到妥善处理。全面推进法治朔州建设，深入开展法治宣传教育，我市被评为全国“六五”普法中期先进市。加强社会治安综合治理，完善社会治安防控体系，刑事案件、治安案件、“两抢一盗”案件大幅下降，有力地维护了社会和谐稳定。与此同时，加强国防后备力量建设，积极发展妇女儿童、老龄和残疾人事业，宗教、人防、地震、应急、气象、档案、地方志等各项工作都取得新成绩。

推进法治政府、廉洁政府建设。认真执行人大及其常委会的决议决定，积极支持人民政协履行职能，全年共办理人大代表建议、政协委员提案204件。支持各民主党派、工商联、无党派人士参政议政，支持工会、共青团、妇联等人民团体发挥作用。认真开展党的群众路线教育实践活动和学习讨论落实活动，严格落实中央八项规定精神，严格执行国务院“约法三章”，加强纪律作风整顿，加大“四风”治理力度，深入推进廉政建设和反腐败斗争。大幅减少了“三公”经费，严肃查处了一些领导干部违纪违法问题。深化“看作为、比建树、争一流”活动，干部作风和部门行风进一步改进。

各位代表，过去的一年，是极不平凡的一年，是经济发展异常艰难的一年。我们攻坚克难、砥砺奋进，经济社会发展取得这些成绩，实属不易。这是省委、省政府和市委正确领导的结果，是市人大、市政协大力支持和有效监督的结果，是全市人民齐心协力、共同奋斗的结果。在这里，我代表市人民政府，向全市人民和所有关心支持朔州发展的各界人士，表示崇高的敬意和衷心的感谢!

在肯定成绩的同时，我们也要清醒地看到，我市经济发展处于近年来最困难的时期。煤炭市场持续低迷，工业企业效益下滑，投资增长后劲不足，经济增速大幅放缓。一煤独大的产业结构没有改变，非煤产业没有壮大起来，发展方式粗放，经济结构不优，质量效益不高。农业基础薄弱，农民增收难度大，农村脱贫任务繁重。城乡基础设施不够完善，社会事业欠账较多，公共服务能力薄弱，民生改善压力较大。安全生产形势不容乐观，社会矛盾积累较多。政府工作还存在不足，有些政策措施没有落实到位。一些政府机关工作人员为官不为、懒政怠政，精神状态不佳。一些领域腐败问题比较严重，党风廉政建设和反腐败斗争任务艰巨。对此，我们一定要高度重视，认真加以解决。二、2015年工作安排

2015年，是全面深化改革的关键之年，是全面推进依法治国的开局之年，是全面完成“十二五”规划的收官之年。

当前，宏观经济形势依然复杂严峻，经济下行压力持续加大，我市今年面临的困难比去年还要大。同时，我们也要看到有利条件和重要机遇。我国发展仍处于重要战略机遇期，经济发展保持中高速增长，煤炭作为我国主体能源的地位不会改变。中央密集出台稳增长调结构政策措施，我省综改试验区建设不断深入，“六大发展”加快推进，新的发展活力不断释放。我市作为全省唯一的成长型资源型城市，被国家有关部门确定为全国工业绿色转型发展试点城市、资源综合利用“双百工程”示范基地、工业固废综合利用基地建设试点、国家草牧业发展试验试点。省委省政府作出在我市布局综合能源基地、晋北煤化工基地、雁门关生态畜牧经济区等重大战略决策。只要我们准确把握新常态，趋利避害、攻坚克难，顺势而为、乘势而上，就一定能够取得推进“两大任务”的新突破新成效，让朔州经济走上良性发展轨道。

今年政府工作的总体要求是：高举中国特色社会主义伟大旗帜，以邓小平理论、“三个代表”重要思想、科学发展观为指导，全面贯彻党的十八大和十八届三中、四中全会精神，贯彻落实习近平总书记系列重要讲话精神和“四个全面”战略布局，按照省委“五句话”总要求，主动适应经济发展新常态，坚持稳中求进工作总基调，紧扣优化经济结构和提升发展质量“两大任务”主题，着力改革攻坚，突出创新驱动，更加注重民生，深入实施“六权治本”，加快推进“六大发展”，促进经济平稳健康发展和社会和谐稳定。

今年经济社会发展的主要预期目标是：地区生产总值增长6%左右，规模以上工业增加值增长6%，全社会固定资产投资增长16%，社会消费品零售总额增长10%，公共财政预算收入降幅控制在15.27%以内，城镇常住居民人均可支配收入增长7%，农村常住居民人均可支配收入增长8%。约束性指标完成省下达任务。

确定这样的预期目标，坚持了积极作为和实事求是的原则，考虑了完成“十二五”规划任务和全省发展对朔州的要求，考虑了预期目标和预算稳健相兼顾，考虑了稳定经济总量和优化经济结构相协调。实现这些目标，必须付出艰苦努力。

主要工作任务是：

(一)扭住稳定增长这个当务之急，全力促进经济企稳回升

解决朔州发展中积累的矛盾和问题，必须把稳增长作为首要任务，必须保持一定的增长速度，必须保住并做大经济总量，这是发展之基、当务之急、根本所在。

努力稳定煤炭生产。稳增长，核心在稳煤炭。一要稳产量。实现经济总量稳定增长，必须有一定的煤炭产量支撑。今年的煤炭产量原则上要维持去年的生产水平。中煤平朔要从大局出发，发挥好龙头作用。对经过技术改造生产能力有明显提升、确实具备安全保障能力的地方煤矿，支持取得合法生产手续，稳定煤炭生产能力。二要提品质。用好煤层配采审批权下放政策，优化煤层开采顺序，促进科学配煤，提高产品质量和市场竞争力。三要保市场。落实电煤长期稳定供应合作协议，探索建立煤炭产销区域联动机制，全力保量保价，努力扩大市场份额。

加大帮扶企业力度。企业是稳增长的根基。认真落实国家和省减轻企业负担促进工业稳定运行的各项政策措施，帮助企业渡过难关。统筹用好各类政府性基金和专项扶持资金，探索开展“助保贷”工作，壮大政府性融资担保公司，促进银企对接，缓解中小企业融资难问题。落实好减免行政事业性收费、缓缴社会保险费用等措施，坚决清理不合理收费，降低企业生产成本。实施中小微企业成长工程，加强帮扶、指导和服务，创优企业发展环境。

狠抓投资增长和项目建设。必须把投资作为稳增长的关键，稳定投资规模，优化投资结构，培育更多新的经济增长点。扎实开展“项目提质增效年”活动，按照省政府开工一批、争取一批、签约一批、招商一批的要求，加大固定资产投资和重点工程建设力度。今年全社会固定资产投资计划安排946亿元，实施省市重点工程226项，总投资2507亿元，年度计划完成投资686亿元。围绕电力统筹发展、循环经济发展、重大基础设施建设、城乡人居环境改善等领域开展重大工程攻坚，着力抓好晋北煤化工基地、山阴昱光二期2×35万千瓦低热值煤发电项目、固废工业园区、应县恒天然奶牛牧场群、平鲁至右玉高速公路、七里河生态环境综合治理等总投资952亿元的二十个标志性工程，今年完成投资168亿元。

突出抓好产业投资，进一步完善新兴产业投资促进机制，继续落实市直部门与县区结对子引进建设投资5亿元以上产业转型项目的要求，把投资的主要力量集中到非煤产业领域。做实“朔州企业家投资在朔州”活动，鼓励有实力的企业家把更多的资金投到转型项目上，激发民间投资活力。积极对接国家“一带一路”、京津冀协同发展战略，主动融入环渤海经济圈，加大招商引资力度，创新招商方式，实施精准招商，突出产业链招商、园区招商，承接更多产业转移。着力抓好基础设施和公共事业投资，发挥政府投资的引导作用。拓宽投融资渠道，推动金融机构增加信贷投放，推广政府与社会资本合作建设模式，破解建设资金紧缺难题。积极对接国家和省重点工程建设计划，继续推动朔州机场、大西高铁朔州段等重大项目建设。

更加注重促进消费增长和出口增长。积极落实各项惠民政策，增加城乡居民收入，增强居民消费能力。以全国第二批中小商贸流通企业服务体系建设试点工作为抓手，完善商业网点规划布局，改善城乡消费环境，促进服务业发展。加快发展电子商务。积极组织参加“山西品牌中华行”“山西品牌丝路行”等专题促销活动，扩大我市名优特产品市场影响。加强外贸工作，努力扩大出口。支持怀仁县、应县加强日用陶瓷外贸转型升级示范基地建设，推动陶瓷出口增长。

(二)把牢优化经济结构这个核心任务，加快提质增效升级

坚持稳增长和调结构相结合，以建设全国工业绿色转型试点城市、工业固废综合利用基地和资源综合利用“双百工程”示范基地为主要抓手，加快资源型经济转型步伐。

推动传统产业转型升级。着力推进煤炭产业向市场主导型、清洁低碳型、集约高效型、延伸循环型、生态环保型、安全保障型转变，努力走出具有朔州特色的“革命兴煤”路子。一要在煤炭产业链延伸上取得新突破。聚焦国家新型综合能源基地建设，进一步延伸煤电、煤化工产业链。加快建设现代化矿井，年内新增标准化矿井5座。加快推进总装机规模258万千瓦的26个在建电厂的建设进度，年内电力装机容量突破1000万千瓦。大力推进蒙西至天津南1000千伏、晋北至江苏800千伏两条特高压电力外送通道建设。加快发展风能、太阳能、生物质能发电项目，提高清洁能源比重。积极推进晋北现代煤化工基地建设，争取阳煤集团80亿立方煤制气、60万吨煤制烯烃等项目早日开工。二要在煤炭资源循环利用上取得新突破。加快形成煤矸石综合利用发电、煤矸石综合利用新材料、粉煤灰资源化开发利用、脱硫石膏综合利用四大产业体系，构建循环经济发展促进机制、资源产业与环保产业协同发展机制，进一步提高煤矸石发电装机比重、工业固体废弃物综合利用率，创出全国煤电基地结构调整和工业固废高效利用新模式。

加快促进新兴产业发展壮大。以文化旅游、装备制造、新能源、新材料、节能环保、日用陶瓷、食品医药、现代服务业八大非煤产业为重点，培育一批主导产业。突出抓好文化旅游业，落实财税、土地、价格等支持政策，建设文化产业示范园区，开发广武边塞文化旅游区，提升杀虎口等旅游景区品质，打造朔州旅游品牌。大力发展以煤机装备为重点的装备制造业，从设备维修、配件加工向成套设备发展，使装备制造业成为我市工业经济的主力军。推动陶瓷业兼并整合，壮大骨干企业，提高新产品新技术研发能力，打造知名品牌，增强市场竞争力。食品医药产业要壮大乳品、酒类、小杂粮、肉食加工等特色产业，发展生物制剂、新型药物制剂。我市节能环保市场巨大，要高度重视发展节能环保产业，努力把节能环保产业打造成新兴支柱产业。

提升开发区和工业园区发展水平。开发区和工业园区要创新体制机制，明确发展定位，完善基础设施，优化服务环境，引进更多企业，壮大经济实力。开发区要努力向中高端迈进，打造全市高新技术产业集聚区。固废工业园区要加快完善基础设施，继续支持北大研发中心建设，新引进两到三家有标志性意义的骨干企业，建设成为全国粉煤灰综合利用示范基地。朔城区富甲工业园区，要强化装备制造、新材料产业的主导地位，加强与中煤等大企业合作，新引进几家煤机装备等制造业企业，建设制造业集聚区。东露天循环经济园区、平鲁北坪工业园区、山阴北周庄工业园区，要以高端化、全循环、链条式发展为主攻方向，建成一批煤电一体、煤化一体新项目，打造全省煤基循环产业示范园区。怀仁金沙滩陶瓷工业园区要引导企业联合整合，推动产品提档升级。怀仁医药工业园区要加大招商力度，积极承接产业转移项目，争取再引进几家医药企业，形成集聚效应，努力发展成为全省重要的医药生产基地。应县新型产业科技园区要积极引进高新技术，发展新能源、高端陶瓷等产业。右玉梁威工业园区要突出生态绿色，下工夫新建一批特色农产品深加工项目，形成全市特色农产品加工示范区。

(三)大力实施雁门关生态畜牧经济区领头雁工程，加快推进农业现代化

以雁门关生态畜牧经济区核心区建设和领头雁工程为统领，坚持以农载牧、以牧富民，加快转变农业发展方式，调整优化农业结构，构建具有朔州特色的现代农业体系，使农业更强、农民更富、农村更美。

大力推进农业产业化。突出发展现代畜牧业。以国家草牧业发展试验试点市工作为抓手，大力推进耕地种草，改造提升天然草场，建设大草场大牧场，为现代畜牧业发展提供有力支撑。三年之内，建设100万亩人工草场和100万亩优质天然牧场。继续实施规模健康养殖工程，年内改造20个奶牛园区、30个肉羊园区。支持应县恒天然奶牛牧场群建设。稳定发展粮食生产。加强农田水利建设，完成桑干河灌区三干渠、四干渠节水改造，新建膜下滴灌节水面积1.1万亩。实施百万亩水地玉米地膜覆盖、玉米丰产方建设、小杂粮提质增效、马铃薯增收致富等高产创建工程，建设220万亩优质玉米基地、50万亩荞麦燕麦等有机杂粮基地、50万亩优质马铃薯基地。促进农业一二三产业融合发展。加快发展农产品加工龙头企业，培育壮大一批引领经济发展的领军企业。加强品牌创建，努力打造一批在全省全国叫得响的特色农产品品牌。积极开拓市场，举办好雁门关生态畜牧经济区特色农产品展销暨技术交流招商引资洽谈会。全年农产品加工企业销售收入要突破180亿元。加快完善农产品流通体系，着力打造雁门关农产品物流园区，加强农产品批发、仓储和冷链等设施建设。加快发展农村旅游，积极开发观光农业、休闲农业、森林旅游、林下经济等产业，培育农民增收新的增长点。

大力推进农业经营规模化。继续推进“一村一品、一县一业”建设，发展多种形式适度规模经营。支持种养大户、家庭农场、农民合作社等新型主体发展，建设100个示范合作社和100个示范家庭农场。加快推进农业机械化，新建10个农机专业合作社，发展10个2000亩以上的全程机械化示范农场。加快构建农业科技创新服务体系，加强以农技站、畜牧兽医站为重点的基层服务机构建设，筹备建设雁门关生态农业研究院。积极推进供销社改革，组建一批高标准的农业综合服务社，完善农业社会化服务体系。加强农产品质量安全监管能力建设，全面开展食品安全县和农产品质量安全县创建工作。

深化农村改革。做好农村土地承包经营权确权登记颁证工作，稳定和完善农村土地承包关系。建设市级农村产权交易市场、县区农村土地流转服务中心、乡镇土地流转服务工作站，有序推进农村土地流转。全面开展农村宅基地确权工作。继续推进集体林权制度改革和草原确权。启动农村集体产权制度改革试点。加强金融支农服务，扩大政策性保险覆盖面，探索开展设施农业和基础母羊保险。

不断改善农村人居环境。扎实开展农村“人居环境改善年”活动。全面推进完善提质、农民安居、环境整治、宜居示范“四大工程”，继续办好“五件实事”。改造农村困难家庭危房6200户，新建改建农村幼儿园13所，易地搬迁农村贫困人口3248人，深入推进乡村清洁工程，培训新型职业农民1万人。针对农村环境脏乱差问题，重点抓好以“三拆、四改、五化”为主要内容的环境整治和清洁工程，做到“垃圾出村、杂物进院、畜禽圈养、人畜分离、街道清洁、村容整洁”。年内，全市90%以上的乡镇要建成环境整治达标乡镇，80%以上的村庄要建成环境整治达标村。

持续打好扶贫攻坚战。实施扶贫攻坚计划，加大资金投入，按照精准扶贫要求，抓好干部驻村帮扶工作，扎实开展易地搬迁、整村推进、产业扶贫、金融扶贫，加快贫困地区脱贫步伐,确保农村贫困人口与全市人民同步进入小康社会。特别是要深入推进集中连片贫困地区扶贫开发，集中资源、集中力量,精准发力、精准脱贫。难度再大，今年也要再减少40个贫困村，完成1.8万贫困人口脱贫任务。

(四)大力加强城市建设，积极推进新型城镇化

城镇化是解决城乡差距的根本途径，是经济增长的最大引擎。要坚持以人为核心，积极推进特色新型城镇化，努力建设彰显自然生态精致宜居现代化城市魅力的塞上明珠。

继续推进“五城联创”。“五城联创”是城镇化的引领工程，要持续用力推进。今年总的要求是，推动创建工作向更高水平迈进，全面启动申报国家级城市创建工作。巩固国家园林城市创建成果，进一步提升园林绿化水平,确保复查验收顺利通过。在成功创建省级双拥模范城市的基础上，力争获得全国双拥模范城市命名。统筹推进国家卫生城市和国家环保模范城市创建工作。巩固省级卫生城市创建成果，广泛开展城乡爱国卫生清洁运动，启动申报国家卫生城市创建工作，完成国家环保模范城市创建工作年度任务。巩固连续三届省级文明城市创建成果，实施创建全国文明城市三年工作规划，在2015年全国城市文明程度指数测评中取得好成绩，为创建全国文明城市打好基础。

加快提高中心城市建设和管理水平。坚持规划引领、生态支撑、文化塑造，加快建设设施配套、功能完善、环境优美、管理先进、宜居宜业的城市人居环境。今年着力抓好十大工程。一是规划修编工程。确定总规，编制详规，统筹布局，强化管控，健全规划管理体制机制。年内完成城市总体规划修编和控制性详细规划编制工作。开展地下管线普查，建立地下管线数据库。二是继续推进朔州老城改造完善工程。三是建成恢河大桥，年内实现通车。四是继续推进七里河市区段综合治理工程。推进路桥建设，实施蓄水、绿化。五是继续推进城中村改造工程。六是完善优质公交工程。继续推进公交体制改革，建设公交站场、调度中心、加油站、加气站，投放新的节能环保公交车，逐步建成现代化、智能化的城市优质公共交通运营服务体系。七是供水工程。改造提升现有水厂，建设第四自来水厂。八是污水垃圾处理工程。建设第二污水处理厂和垃圾处理城南填埋区。九是供热工程。关停中心城区所有供热燃煤小锅炉，实现热电联产集中供热全覆盖。十是绿化工程。重点建设民福街西延线道路绿化等工程。

积极实施大县城战略。推进以大县城为基础的就地城镇化。规划实施平鲁区与中心城区同城化工程。支持山阴县加大县城改造力度，尽快改变县城面貌。怀仁县要进一步提高城镇化水平。应县要依托木塔，建设旅游文化名城。右玉县要发挥生态优势，打造北方避暑胜地。继续实施保障性安居工程，新建各类保障性住房13396套，完成投资30亿元。推进智慧城市建设，保护传承历史文化，加强供水供气供电、公交和污染治理等公共服务设施建设，全面提高县城管理水平。

加强交通运输体系建设。开工建设平鲁至右玉高速公路，推进环城高速东北环前期准备工作。改造干线公路，建设109国道右玉县城改线段，改造平朔一级公路平鲁段。发展旅游公路。实施村通水泥(油)路完善提质工程。加强县乡公路养护，保障道路安全畅通。

(五)全面发展社会事业，切实保障和改善民生

着眼保基本、兜底线、促公平、可持续，加大惠民政策力度，加快发展社会事业，健全基本公共服务体系，增进民生福祉。

着力促进创业就业。坚持就业优先，以创业带动就业。深入推进国家级创业型城市创建活动。加强就业指导和创业培训，突出抓好高校毕业生就业创业工作。继续实行政府购买基层公共服务岗位，吸纳高校毕业生就业。统筹做好退役军人、农村转移劳动力、城镇困难人员等就业工作。积极发展服务业等劳动密集型产业，扶持中小微企业，开发更多就业岗位。鼓励支持困难企业采取在岗培训、协商薪酬等办法，稳定就业岗位。完善创业扶持政策，强化创业孵化基地建设，开展创业小额担保贷款工作。完善就业服务体系，加强乡镇劳动保障机构建设。

努力促进城乡居民增收。进一步调整最低工资标准，完善并落实工资支付规定，保障职工特别是农民工按时足额领到工资报酬，构建和谐劳动关系。落实机关事业单位工资增长政策和县以下机关公务员职务职级并行制度。农村每户一吨冬季取暖用煤改为发放现金。

提高社会保障水平。进一步完善覆盖全民、统筹城乡的社会保障体系。落实机关事业单位养老保险改革措施。继续提高企业退休人员基本养老金、城乡居民基础养老金、城乡低保、农村五保对象补助水平，城乡居民基本医保和新农合年人均财政补助标准由320元提高到380元。实施社会化养老示范工程，鼓励社会力量兴办养老机构，加快发展养老服务业。落实社会救助暂行办法，做好残疾人就业援助、康复救助，全面实施临时救助制度。

推动教育均衡发展。贯彻落实国务院三年行动计划和我省新三年行动计划，加快发展学前教育。加强义务教育学校标准化建设，全面推进国家义务教育均衡发展县建设工作。加强市区中小学校建设，缓解班容量大的问题。继续实施好学生饮用奶工程。加快发展职业教育，加强特殊教育。积极发展高等教育，提升朔州师专和朔州职业技术学院办学水平，支持办好中北大学朔州校区。

提升医疗卫生服务水平。加大公共卫生投入，人均基本公共卫生经费财政补助标准由35元提高到40元。启动城乡居民大病保险制度。完善城乡医疗卫生服务体系，提升县级医院综合服务能力，加强乡镇卫生院建设。为农村60岁以上老年人实行免费体检。做好人口计生工作，推进计划生育服务管理创新。

加强文化建设。提升公共文化服务能力。完成创建国家公共文化服务体系示范区各项任务，加快建设文化强市。加强文化队伍建设，完善文化服务网络，推进公共文化服务均等化。实行政府购买公共文化服务，大力实施文化惠民工程，让人民群众享受更多更好文化发展成果。加强文物和非物质文化遗产保护。广泛开展全民健身活动，提高竞技体育水平。

加强精神文明建设。坚持以人为本、重心下移，综合运用教育引导、文化熏陶、实践养成、制度保障等手段，深入开展精神文明创建活动，培育和践行社会主义核心价值观，发挥法治文化、红色文化、廉政文化的资政育人作用，加强社会公德、职业道德、家庭美德和个人品德教育，树立良好社会风尚。

着眼于解决群众最关心最直接最现实的利益问题，突出抓好十件民生大事。一是启动新建市一中高中部。二是新建市人民医院(朔州大医院)。三是逐步配齐乡镇卫生院医务人员。四是成功创建国家公共文化服务体系示范区。主要任务是加强县乡文化服务设施建设，实现县区图书馆、文化馆达标，提升乡镇文化站服务能力。五是向高校毕业生提供1000个公共服务岗位。六是继续实施农村饮水解困工程。解决59个村2.07万人的饮水困难。七是实施农村安居工程。统筹规划采煤沉陷区治理、地质灾害治理、农村危房改造、扶贫易地搬迁，搬迁36个村12700户。八是全面实施城乡环境卫生清洁工程。九是开展全民参保登记，推动全民参保。十是推进五保对象集中供养。

(六)加强生态文明建设，持续改善生态环境

牢固树立生态文明、绿色发展理念，持之以恒推进环境保护和生态建设，努力建设美丽朔州。

抓好节能减排。推进工业、建筑、交通、公共机构等领域节能降耗，做好煤炭、电力、冶金、化工、建材等重点行业节能工作。强化节能目标管理，逐步建立企业能耗在线监测体系，严格控制能源消耗。狠抓减排治污，强化大气污染防治。深入开展重点行业脱硫、脱硝、除尘改造，推进燃煤电厂超低排放改造，抓好农业源减排工作。市县都要推广集中供热，减少燃煤锅炉。今年，二氧化碳、化学需氧量、氨氮、氮氧化物等各项排放指标继续降低。

持续推进造林绿化。落实省政府“三加三不减”要求，全年营造林22万亩。重点抓好朔城区西山环城绿化八期、平鲁区龙雨山荒山造林、山阴县西山生态综合治理、怀仁县西山生态经济园林区绿化、应县龙首山造林绿化和右玉县大南山荒山造林六大工程。按照“一乡一条路、一村一片林、人均五棵树”的要求，搞好乡村绿化。加强森林管护，巩固绿化成果。

加强水生态建设。实行最严格的水资源管理制度，节约集约利用水资源，实现水资源的可持续开发利用。抓好重要水利工程建设，积极推进七里河上游综合治理工程和太平湖治理，开工建设应县小石口水库、怀仁县东作里水库。做好防汛防洪保安工程，建设东榆林水库专项应急除险加固工程和山阴县黄水河河道治理工程。

积极推进生态治理修复。健全生态补偿机制，开展采煤沉陷区、采空区、煤矸石山的土地复垦和生态治理，防治地质灾害。

(七)引深转型综改区建设，推动改革创新实现新突破

以综改试验区建设为统领，认真实施“224”年度行动计划，突出抓好重点领域和关键环节改革，创新体制机制。

推进矿产资源市场化改革。对新设立的矿业权全部采用招标、拍卖、挂牌等市场竞争方式出让，完善矿业权二级交易市场。严格落实省政府煤炭领域改革部署，促进煤炭工业可持续发展。深化户籍制度改革。全面放开落户条件，统一城乡户口登记制度。全面实施居住证制度，对在城镇就业居住但未落户的外来人口，以居住证为载体提供相应的基本公共服务，推进城镇基本公共服务常住人口全覆盖。推进公共资源交易平台建设。规范完善市级公共资源交易平台。对需要进入平台进行交易的公共资源，坚持应进必进，坚决杜绝场外交易。深化财税体制改革。完善政府预算体系，将政府收支全部纳入预算管理，未列入预算的不得支出。加强财政资金绩效管理，盘活财政存量资金。加强政府债务管理。推进金融改革发展。推动农村信用社改革，筹建朔州市农村商业银行。鼓励发展民营银行和村镇银行，促进融资性担保企业、小额贷款公司健康发展。创新金融服务，开展贷款保证保险试点工作，扶持实体经济发展。发展资本市场，加强企业上市培育力度。优化金融生态，防范和打击非法集资活动，维护金融秩序稳定。深化教育资源配置机制改革。推行教师轮岗交流制度，在市区内逐步实行教师“局管校用”管理制度，促进教育资源合理配置。深化医药卫生体制改革。全面推进县级公立医院改革，完善基本药物制度和基层运行新机制，进一步规范药品流通秩序。深化文化体制改革。进一步理顺政府部门与所属文化企事业单位关系，推动文化事业单位组建理事会，推进市属国有文艺院团转企改制工作，培育文化产业投资主体，活跃文化市场。

推进科技创新。实施国家创新驱动发展战略朔州行动计划和朔州低碳创新行动计划。加强科技创新体系建设，建立产学研协同创新机制。强化企业在科技创新中的主体地位，支持企业建设技术研发机构。筹备建设科技创新和大学生创业园区，培育和引进高新技术企业。加强以煤基科技、固废综合利用、特色现代农业为重点的科技攻关。保障科技投入，严格实施市级财政科研项目和资金管理办法，提升科技资金使用绩效。

(八)扎实抓好安全稳定工作，促进社会和谐

安全稳定是最大的民生，是比泰山还重的责任。必须高度重视，警钟长鸣，常抓不懈，为全市经济社会发展奠定坚实基础。

毫不松懈抓好安全生产。牢固树立安全发展理念。坚持任何时候都决不能过高估计安全生产形势、决不能过高估计干部群众对安全生产重要性的认识、决不能过高估计我们保证安全生产的能力和水平，时刻敬畏生命、敬畏责任、敬畏制度。加强安全生产责任体系建设。严格落实新修订的安全生产法，坚持“党政同责、一岗双责、齐抓共管”，落实政府安全监管责任，强化企业安全生产主体责任。按照“行业主管部门直接监管，安全监管部门综合监管，地方政府属地监管；管行业必须管安全，管业务必须管安全，管生产经营必须管安全”的要求，全面加强煤矿、道路交通、非煤矿山、冶金工贸、危险化学品、水库、人员密集场所以及建筑施工、油气管道、城市燃气、特种设备等各行业各领域的安全生产监管工作。推进企业安全生产标准化建设，提高现场管理水平，加强应急救援能力建设，夯实基层基础。深入开展隐患排查治理，严厉打击非法违法建设生产经营行为。强化考核问责，严格落实安全生产目标责任考核“一票否决制”，严肃查处事故，严格追究责任，坚决杜绝重特大事故、减少一般性事故。

加强和创新社会管理。深入开展法治宣传教育，提高全民法治意识。落实重大决策社会稳定风险评估机制，有效预防和化解社会矛盾。把信访纳入法治轨道，及时就地解决群众合理诉求，开展信访积案百日攻坚。深化平安朔州建设，加强社会治安综合治理，完善立体化治安防控体系，深入开展“打黑除恶”斗争，提高人民群众安全感。支持国防后备力量建设。

(九)坚持依法行政，建设法治政府和廉洁政府

认真贯彻落实党的十八届四中全会精神，实施“六权治本”，加快建设职能科学、权责法定、执法严明、公开公正、廉洁高效、守法诚信的法治政府。

深入推进政府职能转变。深化政府机构改革，推进机构、职能、权限、程序、责任法定化，做到法定职责必须为、法无授权不可为。年底前编制完成市级政府部门权力清单、责任清单，明年年初公布运行。深化行政审批制度改革，加大简政放权力度，进一步取消、下放行政审批事项，全面清理非行政许可审批。厘清市区两级政府职权，理顺城管执法体制。深化社会组织管理制度改革，推进行业协会商会与行政机关脱钩。

强化权力运行制约监督。健全依法决策机制，把公众参与、专家论证、风险评估、合法性审查、集体讨论决定作为政府重大行政决策的法定程序。严格权力制约，落实单位主要负责人“五个不直接分管”规定。建立重大决策终身责任追究制度及责任倒查机制。全面推进政务公开，抓好政务服务平台和公共资源交易平台建设。

加强反腐倡廉建设。从严落实政府系统党风廉政建设责任制和党委(党组)主体责任，严格落实“一岗双责”。认真贯彻落实中央八项规定精神，严格执行国务院“约法三章”，持续狠刹“四风”。厉行节约，反对浪费，严控“三公”经费，推进公务用车改革。加强行政监察和审计监督，对公共资金、公共资源、国有资产严加监管。始终保持反腐高压态势，扎实开展重点领域和关键环节专项整治，努力铲除滋生腐败土壤。

科学编制“十三五”规划。以改革的精神、创新的理念和科学的方法，紧扣“两大任务”主题，把握“六大发展”方向，深入调查研究，充分谋划论证，做好“十三五”规划编制工作，描绘好未来五年发展蓝图。

狠抓工作落实。经济发展进入新常态，精神面貌要有新状态。越是困难的时候，越要振奋精神。要巩固党的群众路线教育实践活动和学习讨论落实活动成果，进一步抓好整改落实，完善长效机制。认真开展“三严三实”专题教育，大力弘扬右玉精神，以扎实过硬的作风，强力推动各项工作。深化“看作为、比建树、争一流”活动，营造干事创业浓厚氛围。深入开展纪律作风整顿，对不担当、不作为、乱作为的干部，坚决严肃处理。强化督查考核，确保各项决策部署落到实处，确保各项目标任务顺利完成。广大公务员特别是领导干部要始终把为人民谋福祉作为最大责任，始终把改革发展稳定的使命扛在肩上，始终保持积极向上的精神状态，履职尽责，敢于担当，奋力作为，不辱历史使命，不负人民重托!

各位代表，当前朔州正处于转型发展、创新发展、爬坡过坎的关键历史时期。让我们紧密团结在以习近平同志为总书记的党中央周围，在中共朔州市委的坚强领导下，坚定信心、排除万难，团结拼搏、开拓奋进，为开创弊革风清、富民强市的新局面，实现全面建成小康社会的宏伟目标而努力奋斗!
